# Moderate Stress Level among Patients Undergoing Hemodialysis in a Tertiary Care Centre

**DOI:** 10.31729/jnma.8231

**Published:** 2023-08-31

**Authors:** Manoj Dhungana, Pragya Shrestha, Sundar Pandey, Sreya Paudyal, Krishna Kumar Agrawal

**Affiliations:** 1 Department of Psychiatry, Devdaha Medical College and Research Institute, Bhaluhi, Rupandehi, Nepal; 2 Department of Nursing, Universal College of Medical Sciences, Bhairahawa, Rupandehi, Nepal; 3 Department of Internal Medicine, Devdaha Medical College and Research Institute, Bhaluhi, Rupandehi, Nepal; 4 Department of Psychiatry, Universal College of Medical Sciences, Bhairahawa, Rupandehi, Nepal; 5 Department on Internal Medicine, Universal College of Medical Sciences, Bhairahawa, Rupandehi, Nepal

**Keywords:** *hemodialysis*, *prevalence*, *psychiatric disorders*

## Abstract

**Introduction:**

Stress is the response of body to any change. The end stage of renal disease and the process of haemodialysis treatment are long-term stressors that alter patients' well-being and everyday lifestyle. The aim of this study was to find out the prevalence of moderate stress levels among patients undergoing hemodialysis in a tertiary care centre.

**Methods:**

A descriptive cross-sectional study was conducted among patients undergoing hemodialysis from 1 October 2021 to 30 September 2022. Ethical clearance from the Institutional Review Committee. Patients undergoing maintenance haemodialysis for at least 3 months were included in the study. Convenience sampling method was used. The point estimate was calculated at a 95% Confidence Interval.

**Results:**

Among 118 patients, 82 (69.49%) (61.18-77.80, 95% Confidence Interval) had moderate stress levels, out of which 51 (62.20%) were male and 31 (37.80%) were female.

**Conclusions:**

The prevalence of moderate stess level was found to be higher than other studies done in similar settings.

## INTRODUCTION

Stress can be defined body's response to anything that requires attention or action. As any type of change that causes physical, emotional or psychological strain.^1^

Since dialysis is unable to fully compensate for the metabolic activities of people, this is considered one of the potential stress factors among dialysis patients.^2^ The dialysis patient due to the aggravation of chronic renal failure, affects the psychosocial function of these people, in a way that many psychiatric disorders are seen in these patients, including stress, depression and anxiety.^3^

The aim of this study was to find out the prevalence of moderate stress level among patients undergoing hemodialysis in a tertiary care centre.

## METHODS

A descriptive cross-sectional study was conducted among the patients admitted to Devdaha Medical College and Research Institute, Bhaluhi, Rupandehi, Nepal from 1 October 2021 to 30 September 2022. Ethical approval was taken from the Institutional Review Committee (Reference number: 23/2021). Patients undergoing maintenance haemodialysis for at least 3 months and those who were willing to participate in the study were included. Non-compliant patients with known cognitive and mental impairments and those under 18 years old were excluded from the study. Convenience sampling method was done. The sample size was calculated by using the following formula:


$$\begin{array}{ll} \text{n} & ={\text{Z}}^{2}\times \frac{\text{p}\times \text{q}}{{\text{e}}^{\text{2}}}\\ & =1.96^{2}\times \frac{0.50\times 0.50}{0.10^{2}}\\ & =96 \end{array}$$

Where,

n = minimum required sample size
Z = 1.96 at 95% Confidence Interval (CI)
p = prevalence taken as 50% for maximum sample size calculation
q = 1-p
e = margin of error, 10%

The minimum required sample size was 96. However, 118 patients were taken for the study. The classification of stress was done as low stress, moderate stress, and high perceived stress based on the perceived stress scale.^6^ The data were collected by using a face-to-face interview and the patient's treatment record report.

Data were entered in Microsoft Excel and analysed using IBM SPSS Statistics version 16.0. The point estimate was calculated at a 95% Confidence Interval.

## RESULTS

Among 118 patients, 82 (69.49%) (61.18-77.80, 95% CI) patients had moderate stress levels undergoing haemodialysis.

**Table 1:** Socio-demographic characteristics (n = 82).

Variables	n (%)
**Age (years)**	
18-20	3 (3.66)
21-39	12 (14.63)
40-59	42 (51.22)
60 and above	25 (30.49)
**Sex**	
Male	51 (62.20)
Female	31 (37.80)
**Residence**	
Urban	30 (36.59)
Rural	52 (63.41)
**Education status**	
Illiterate	21 (25.61)
Basic education	32 (39.02)
Secondary education	26 (31.71)
Higher education and above	3 (3.66)
**Marital status**	
Unmarried	4 (4.88)
Living with spouse	69 (84.15)
Single	9 (10.98)
**Type of family**	
Nuclear	39 (47.56)
Joint	43 (52.44)

Among 82 patients, 71(86.59%) patients had theunderlying co-morbid condition and 5 (6.10%) patientsunderwent hemodialysis treatment for more than 36months (Table 2).

**Table 2:** Duration of hemodialysis treatment (n = 82).

Duration (months)	n (%)
<12	47 (57.32)
12-24	19 (23.17)
25-36	11 (13.41)
>36	5 (6.10)

Seventy (85.37%) patients had caretaker to come to hemodialysis centre.

## DISCUSSION

Among 118 patients, 82 (69.49%) patients had moderate stress levels undergoing haemodialysis which is higher than in similar studies. The finding of the study revealed that only 14% have a moderate level of stress.^5^ In a similar study that was conducted at Krishna Hospital Karad, among 30 patients undergoing hemodialysis only 3% of patients had moderate stress.^6^ Previous study conducted at Patan Hospital, Nepal among 59 hemodialysis patients showed 6.78% had moderate stress.^7^

Among 82 respondents with moderate level of stress, 42 (51.22%) respondents were of 40-59 years, the mean age was 49.87±14.36 years, and 51 (62.20%) were male. In contrast, the findings of the study showed that 77.8% of the patients were male, with an average age of 53.52 years and 66.7% were married.^7^ Similarly the mean age of respondents in another study was 51.27 years while 64.41% of the patients were male.^7^

In our study 32 (39.02%) had basic education while other similar studies showed 76.7% had formal education. In our study 43 (52.44%) respondents stayed in joint family while previous study done showed that 80% stayed in joint family, and 20% had extended family.^6^

The study has some limitations. Since it is a descriptive cross-sectional study conducted in a single centre with a small sample size therefore it cannot be generalized to the whole population.

## CONCLUSIONS

The prevalence of moderate stress level among patients undergoing hemodialysis was found to be higher than in other studies done in similar studies. Though the hemodialysis is a life-long intervention so patients are predisposed to developing many kinds of psychiatric disorders therefore, the necessity of planning and proper interventions is felt by healthcare providers to control stress among dialysis patients to prevent mental illness.
